# Magnetic Resonance Properties of Thin Py Films Suitable for Excitation of Spin Waves

**DOI:** 10.3390/nano16191209

**Published:** 2026-09-24

**Authors:** Tetiana Kalmykova, Sergei Krylov, Pavol Neilinger, Tomáš Ščepka, Jan Derer, Sergey Polevoy, Vladimír Cambel

**Affiliations:** 1 Institute of Electrical Engineering, Slovak Academy of Sciences, Dubravska cesta 9, SK-84104 Bratislava, Slovakia; sergey.krylov@savba.sk (S.K.); tomas.scepka@savba.sk (T.Š.); jan.derer@savba.sk (J.D.); vladimir.cambel@savba.sk (V.C.); 2 Department of Radiospectroscopy, O. Ya. Usikov Institute for Radiophysics and Electronics, National Academy of Sciences of Ukraine, Ac. Proskura St. 12, 61085 Kharkiv, Ukraine; polevoy@ire.kharkov.ua; 3 Department of Experimental Physics, Comenius University, SK-84248 Bratislava, Slovakia; pavol.neilinger@fmph.uniba.sk

**Keywords:** ferromagnetic resonance, spin waves, permalloy, magnonics, spintronics, atomic force microscopy

## Abstract

Low magnetic damping and narrow ferromagnetic resonance (FMR) linewidth are essential for efficient spin-wave excitation in magnonic devices. We investigate how a combination of deposition techniques, interface engineering, and consequent thermal treatment affects magnetic losses in thin permalloy (Py, Ni_80_Fe_20_) films prepared by electron-beam evaporation and magnetron sputtering. Broadband vector network analyzer FMR, atomic force microscopy, and electromagnetic simulations were used to correlate linewidth, surface morphology, and spin-wave excitation. Comparable minimum linewidths were reached by both deposition routes: approximately 23 Oe at 4 GHz for the best electron-beam-evaporated film and approximately 21 Oe for a 20 nm TaN/Py/TaN structure. Electron-beam-evaporated films proved sensitive to substrate choice, surface preparation and—in the absence of plasma cleaning—deposition temperature, whereas the magnetic field applied during growth had only a weak effect. After magnetic annealing, however, only the TaN-encapsulated structure retained its narrow linewidth, while the uncapped films broadened substantially. Simulations further showed that linewidth reduction enhances the excitation efficiency and visibility of standing spin-wave modes. These results indicate that comparable losses are achievable through several process routes, whereas retaining them through thermal processing requires interface engineering—a distinction that matters for integrating Py films into magnonic and spintronic devices.

## 1. Introduction

Magnonic devices employing spin waves as information carriers are considered a promising platform for next-generation microwave electronics because they offer low power consumption and the possibility of wave-based computing [1,2,3,4]. Their practical implementation, however, critically depends on magnetic thin films exhibiting low damping, narrow ferromagnetic resonance (FMR) linewidths, and high structural homogeneity. The FMR linewidth is determined by both intrinsic magnetic relaxation and extrinsic broadening mechanisms and therefore provides an important measure of the dynamic magnetic losses in thin films [5]. Consequently, minimizing these losses is one of the key technological challenges for efficient spin-wave excitation and propagation in magnonic devices.

Among soft magnetic materials, permalloy (Py, Ni_80_Fe_20_) remains the material of choice because of its low coercivity, high magnetic permeability, and relatively small intrinsic damping [1,6,7,8,9]. Nevertheless, the dynamic performance of Py-based magnonic structures can be strongly affected by extrinsic linewidth broadening associated with structural and magnetic inhomogeneities. Surface and interface roughness, grain morphology, defects, local variations of magnetic anisotropy, and two-magnon scattering are among the mechanisms known to contribute to magnetic losses in thin films [10,11,12,13,14]. In nanometer-thick films, where the relative contributions of surfaces and interfaces are particularly important, controlling film growth and interface quality is essential for achieving narrow, reproducible FMR linewidths.

Previous studies have demonstrated the effects of individual technological parameters—including deposition technique, substrate material, deposition temperature, film thickness, interface layers, and post-deposition magnetic annealing on the structural and magnetic properties of Py films [6,8,9,14,15,16,17]. However, these parameters are commonly investigated separately or under different experimental conditions, making it difficult to assess their relative importance when several fabrication parameters are relevant simultaneously. In particular, it remains unclear which technological modifications provide the most effective reduction of magnetic losses when considered within a common fabrication route, and whether improvements obtained by modifying the deposition conditions can be further enhanced by controlling thickness, interfaces, and post-deposition treatment.

This issue is particularly important for practical magnonic structures, where fabrication optimization must balance low magnetic losses with thermal stability and compatibility with multilayer device architectures. Interface engineering using buffer and capping layers has recently emerged as a promising approach for improving interfacial quality, protecting the magnetic layer, and modifying its dynamic magnetic properties [14,17,18]. For Py-based structures, such layers can influence surface morphology and magnetic homogeneity and may therefore affect both intrinsic and extrinsic contributions to the measured FMR linewidth. Nevertheless, a systematic comparison of interface engineering with other fabrication parameters under closely controlled experimental conditions is still lacking.

In the present work, we perform a systematic investigation of thin Py films prepared by electron-beam evaporation and magnetron sputtering, varying the substrate type, deposition temperature, magnetic-field conditions during growth, film thickness, TaN buffer and capping layers, and post-deposition magnetic annealing. Broadband FMR and atomic force microscopy (AFM) are employed to characterize dynamic magnetic response and surface morphology. At the same time, numerical simulations are used to evaluate the influence of the experimentally observed linewidth on spin-wave excitation. Rather than constructing a large experimental matrix to independently investigate every possible combination of fabrication parameters, we use a sequential optimization approach in which selected technological parameters are modified between comparable sample groups. This approach enables the incremental improvement associated with selected technological modifications to be assessed while substantially reducing the number of samples and experiments required. Using this approach, we evaluate the relative importance of deposition technology, film thickness, interface engineering, and thermal treatment for obtaining low-loss Py films and identify a practical route toward thermally stable structures suitable for magnonic applications.

## 2. Materials and Methods

Three series of Py thin-film samples were prepared and investigated. The samples differed in deposition method, substrate type, Py thickness, presence of TaN buffer and capping layers, and post-deposition magnetic annealing conditions. The sample parameters are summarized in Table 1. For all substrates, standard cleaning with acetone and isopropyl alcohol (IPA) in an ultrasound bath was used.

The first series of samples was prepared by electron-beam evaporation using an AJA Orion 8E system (AJA International, Inc., Hingham, MA, USA). The Py films had a nominal thickness of 40 nm and were deposited on Si/SiO_2_ and sapphire substrates. During fabrication, several deposition conditions were varied to evaluate their influence on the magnetic resonance properties of the films. These included deposition temperatures, substrate cleaning procedure, and the magnetic-field environment during deposition. In some cases, deposition was performed at elevated deposition temperatures. In other cases, an external magnetic field was applied near the target, or the sample was shielded from it during deposition. Selected substrates were additionally treated by plasma cleaning before deposition.

The second series of samples was prepared by a custom DC magnetron sputtering setup at room temperature, using the same deposition system as in [19]. This method was used to obtain films with improved thickness uniformity and reduced surface roughness. In this series, both the Py thickness and the use of TaN layers were varied in order to evaluate their influence on the FMR linewidth. Films with Py thicknesses of 20 and 40 nm were prepared on Si/SiO_2_ substrates. One of the samples consisted of a 20 nm-thick Py layer inserted between thin TaN buffer and capping layers. The layers were deposited to a nominal thickness of 2.5 ± 0.5 nm, with the deposition rate calibrated by AFM step-height measurement. The TaN thickness was not varied in this study and was not optimized: the layers were required only to be thick enough to act as a continuous barrier, and sputtered TaN has been shown to remain barrier-effective at thicknesses well below this value [20,21]. The tolerance therefore reflects the specification adopted rather than measured run-to-run variation, and individual layers of 1.2–1.5 nm thickness are routinely deposited with defined control on the same system [19].

The TaN layers were introduced to improve adhesion, stabilize the interfaces, and suppress interdiffusion, as metallic buffer and capping layers such as Ti and Ta are commonly used in thin magnetic-film structures for those purposes. However, TaN was selected in this work because transition-metal nitrides are known for their high thermal stability and efficient diffusion-barrier properties [17]. Reactively sputtered TaN provides better diffusion-barrier performance than elemental Ta of the same thickness [22], and TaN capping layers of comparable thickness have previously been used to protect Py films from oxidation in ferromagnetic-resonance studies [8].

The third series consisted of selected samples from the previous series annealed in a static magnetic field. Magnetic annealing was performed at 200 °C for 2 h in a 6N nitrogen atmosphere and a constant magnetic field of 400 Oe. Magnetic annealing was applied to promote stress relaxation, domain stabilization, and possible uniaxial anisotropy formation. The influence of magnetic annealing was studied by comparing the magnetic-resonance and surface properties of annealed and non-annealed samples with similar layer structures. Samples 1.6, 2.1, and 2.4 were chosen for having the narrowest FMR linewidths.

The dynamic magnetic properties of the Py films were studied by broadband ferromagnetic resonance using a vector network analyzer (VNA FMR, Keysight Technologies, Santa Rosa, CA, USA). During the measurements, the sample was placed in a static external magnetic field, H_dc_, while a microwave probing field, H_ac_, was generated by a coplanar waveguide. The sample was positioned across the waveguide’s central conductor, where the microwave magnetic field was concentrated.

The FMR spectra were measured by sweeping the magnetic field at selected fixed frequencies. The absorption spectra were extracted from the standard transmission parameter ∣S_21_∣ of the microwave line. The ferromagnetic resonance linewidth, ΔH, was determined as the full width at half maximum of the measured absorption spectra at selected frequencies in the range of 4–6 GHz. This interval was selected because it covers the frequencies relevant to spin-wave excitation in the investigated structures and provided a well-resolved resonance for all samples, including the strongly broadened annealed ones.

Each sample was measured with both positive and negative magnetic-field sweeps, and the difference between the two sweep directions was taken as an estimate of the uncertainty of ΔH; it amounts to approximately 5% for the as-deposited films and 7–8% for the annealed ones, and exceeds both the point-to-point scatter of the spectra and the sensitivity of the fitted parameters to the chosen fit interval. Linewidths determined by vector-network-analyzer FMR have been shown to agree with those obtained by stripline FMR and pulsed inductive microwave magnetometry on the same Py films, to within about 5% [5].

The frequency dependence of the measured linewidth was analyzed using the expression ΔH = (4π/γ)·α_eff_·f + ΔH_0_, adapted from [8] to the full width at half maximum, where ΔH_0_ is the frequency-independent (inhomogeneous) contribution and γ is the angular gyromagnetic ratio. For an ideal homogeneous film, the slope corresponds to the Gilbert damping parameter α; in real films it may also contain frequency-dependent extrinsic contributions [15], and the fitted value is therefore denoted the effective Gilbert damping α_eff_. The resulting values are listed in Table 2. The effective magnetization M_eff_ was obtained from the frequency dependence of the resonance field. The corresponding uncertainties are also provided in the Figures.

The magnetic field was monitored using a Hall sensor (AREPOC s.r.o., Bratislava, Slovakia). Calibration of the Hall sensor was performed using 2,2-diphenyl-1-picrylhydrazyl (DPPH) powder (Sigma-Aldrich, Merck KGaA, Darmstadt, Germany), which is a standard electron paramagnetic resonance compound with g = 2.0036. The DPPH powder was attached to the waveguide using adhesive tape. A schematic representation of the experimental setup is shown in Figure 1.

Numerical simulations were performed in CST Studio Suite using the built-in frequency-domain solver. The simulated structure consisted of a Py film with lateral dimensions of 50 × 12 µm^2^ placed on a 20 µm-thick silicon or sapphire substrate. A static magnetic field of 280 Oe was applied in the film plane along the x-axis. Microwave excitation was provided by a microstrip line 1000 µm wide and 600 µm long, positioned on the substrate along the x-axis. A microstrip line was used as a simplified single-conductor excitation geometry. Unlike the coplanar waveguide used in the measurements, it produces a microwave field that is symmetric with respect to the center of the film. This symmetry determines which modes are efficiently excited. The geometry of the simulated structure is shown in Figure 2. The film thickness in each simulation was set to the corresponding experimental value.

The transmission coefficient, |S_21_|, was calculated in the frequency range of 4–7.5 GHz. For each simulated structure, the magnetic parameters of the Py film were taken from the corresponding experiment: the effective magnetization listed in Table 2 and a g-factor of approximately 2. The calculated resonance positions therefore correspond to the measured magnetization of each individual sample, and the differences between the simulated spectra reflect the differences in both linewidth and effective magnetization.

In the simulation, the experimentally measured linewidth was assigned to the magnetic-film material properties at each frequency. The components of the permeability tensor were evaluated internally from this linewidth, assuming a linear frequency dependence of ΔH. The effective Gilbert damping was therefore not an independent input, but followed from the assigned linewidth. Above 6 GHz, beyond the measured range, the linewidth was extrapolated using the same linear dependence.

The simulations were used to calculate the transmission spectra of selected samples and to analyze the spatial distribution of the dynamic magnetic-field component H_z_. This enabled the influence of experimentally measured magnetic losses on the visibility and excitation of standing spin-wave modes in the Py films to be evaluated.

Surface topography was characterized using an NT-MDT NTEGRA Prima Scanning Probe Microscope (NT-MDT BV, Apeldoorn, The Netherlands). AFM measurements were performed using commercial Bruker RTESP-150 probes with a nominal tip radius of 10 nm (Bruker Corporation, Billerica, MA, USA). Magnetic-force microscopy measurements were carried out using MESP-LM-V2 probes with a nominal tip radius of 25 nm from the same company.

The AFM measurements were used to determine the surface roughness (both S_a_, average roughness and S_q_, RMS) of the Py films and to compare the influence of deposition method, film thickness, magnetic annealing, and TaN layers on the film topography. Throughout this study, the surface roughness is discussed in terms of S_a_; the corresponding S_q_ values are listed in Table 2 for reference. The obtained roughness data were then compared with the FMR linewidth values in order to analyze the relationship between surface topography and dynamic magnetic properties. In addition, magnetic force microscopy measurements were performed to confirm the absence of pronounced magnetic domain contrast on the sample surface. No pronounced domain contrast was detected by magnetic force microscopy on any of the samples, indicating the absence of large-scale domain structures that could contribute to inhomogeneous broadening [16]. In addition to the S_a_ surface roughness, the lateral correlation lengths along the x- and y-directions (L_x_ and L_y_) were obtained from autocorrelation analysis of the AFM surface profiles.

## 3. Results and Discussion

The comparison of all investigated Py films shows that the FMR linewidth is strongly affected by the deposition technique, film thickness, interface engineering, and post-deposition thermal treatment. Although each fabrication parameter influences the magnetic properties, their effects are not equivalent. Among the investigated technological modifications, the combination of reduced Py thickness and TaN buffer and capping layers provides the most pronounced reduction in linewidth while simultaneously improving thermal stability. The optimization was performed sequentially. First, the deposition conditions for electron-beam evaporation were compared. This was followed by targeted comparisons of magnetron sputtering, Py thickness, and TaN interface engineering. Finally, the thermal stability of selected low-linewidth structures was assessed by post-deposition magnetic annealing.

To assess the relative importance of each technological factor, the FMR linewidth and surface topography of all investigated films were systematically compared. Because the investigated sample set was designed for sequential technological optimization rather than a full factorial study, the relative importance of the parameters is assessed from controlled sample-to-sample comparisons rather than from a formal statistical decomposition. The measured linewidths are discussed below with respect to the influence of deposition technique, interface engineering, and post-deposition magnetic annealing. The autocorrelation analysis additionally showed substantial variation in the lateral characteristic length scales of the surface morphology among the samples (Table 2). However, no simple one-to-one relationship between lateral correlation length and FMR linewidth was observed, suggesting that the linewidth is not determined by the lateral morphology alone.

For the first series of samples, prepared by electron-beam evaporation, the narrowest linewidth was observed for sample 1.6, consisting of a 40 nm-thick Py film deposited on a sapphire substrate. The use of sapphire as a substrate, together with plasma cleaning before deposition, likely improved the interface condition and contributed to a more uniform magnetic film; such interface and magnetic inhomogeneities are known to influence the FMR linewidth [12,14]. The other samples from the first series showed broader resonance lines in comparison to the sapphire substrate. Samples 1.1–1.5 were used to probe individual technological factors. Deposition at 200 °C without plasma cleaning (1.1) produced the broadest line of the series, with the largest inhomogeneous broadening (ΔH_0_ = 9.0 Oe). At the same temperature with plasma cleaning (1.5), ΔH_0_ falls to 5.0 Oe, and the linewidth becomes comparable to that of the room-temperature film (1.4). This indicates that an elevated deposition temperature is detrimental mainly when the substrate surface has not been plasma-cleaned before deposition. At a substantially higher deposition temperature, the effect can no longer be compensated: sample 1.7, deposited at 400 °C with plasma cleaning, showed no measurable FMR response. Such behavior may be associated with substantial microstructural and magnetic changes induced by the high substrate temperature during film growth [24]; irreversible structural changes degrading the magnonic properties of Py have also been reported after thermal treatment at comparable temperatures [23].

The dependence on magnetic shielding (1.2), the applied field during deposition (1.3), and the sample-preparation procedure remained weak, and none of these produced differences comparable to those associated with substrate choice and plasma cleaning (1.6).

This suggests that variations in substrate type, deposition temperature, and magnetic-field conditions during deposition can strongly affect the quality of electron-beam-evaporated Py films. In particular, increased roughness, structural disorder, or local variations in magnetic parameters can enhance extrinsic linewidth broadening [11,12,13].

Sample 1.6 was therefore selected as the optimized electron-beam evaporation reference for subsequent comparison with the other fabrication approaches. Sample 1.7 was excluded from the FMR analysis, since it did not exhibit a measurable FMR response.

Within the sputtered series (the second series), reducing the Py thickness from 40 to 20 nm alone was not found to narrow the FMR linewidth: the uncapped 20 nm film (2.1) exhibited the broadest line of this series. Narrower lines were obtained for the plasma-cleaned 40 nm film (2.3) and for the 20 nm TaN/Py/TaN structure (2.4), whose linewidths differ by about 0.3 Oe, well within the ±5% measurement uncertainty (Section 2, Materials and Methods). The 20 nm thickness was retained for the interface-engineering step, and the distinguishing feature of the TaN structure is not its marginally lower linewidth but its retention of the low-loss behavior after magnetic annealing. Relative to the uncapped 20 nm film (2.1), the narrower line of the TaN/Py/TaN structure is associated with lower surface roughness, improved interface quality, and suppression of extrinsic broadening mechanisms, including two-magnon scattering and local variations in magnetic anisotropy [11,12,13,14]. Although reducing the film thickness may increase the influence of surfaces and interfaces, the use of buffer and capping layers (i.e., TaN) compensates for this effect by improving the structural and magnetic uniformity of the film [8,9,14,17,18]. The TaN-encapsulated structure was therefore selected for the subsequent thermal-treatment study.

It is difficult to identify a clear trend in these parameters as a function of sample number. Yet, within each series, some trends can be traced. Thus, for the first series of samples, the decrease in linewidth with increasing sample number appears to be predominantly associated with the reduction of the inhomogeneous broadening, since α_eff_ varies in both directions without a clear trend. In the second series, the measured linewidth decreases monotonically with sample number, accompanied by a monotonic decrease of α_eff_**,** whereas ΔH_0_ remains within 3.4–5.0 Oe without a systematic trend. In the third series, the linewidth variation after magnetic annealing is dominated by changes in α_eff_.

The values of α_eff_ obtained for the as-deposited films (0.0063–0.0084) lie within the range reported for Py films of comparable thickness (0.004–0.008) [8]. They are somewhat above the intrinsic value of approximately 0.006 obtained for Py after correction for radiative and eddy-current contributions [25], as expected for an effective damping parameter that includes such extrinsic contributions.

A representative ΔH(f) dependence, measured for sample 1.6 over an extended frequency range of 2–14 GHz, is shown in Figure 3 together with the linear fit. The linear behavior is preserved over the whole range, and the parameters obtained from the extended range agree with those from the 4–6 GHz interval used for the systematic comparison (ΔH_0_ = 4.5 versus 4.7 Oe), confirming that the restricted range does not bias the extracted values. All samples showed the same linear dependence within 4–6 GHz (see Figure S1 for the frequency dependence ΔH(f) over the extended range of 3–9 GHz).

The fitted magnetic parameters provide additional insight into the linewidth variations. The effective magnetization M_eff_ varies among the investigated samples, indicating that the fabrication conditions affect the magnetic properties of the Py films. However, the changes in M_eff_ do not show a direct one-to-one correspondence with the measured FMR linewidth. Magnetic annealing, however, produces a systematic change. In both uncapped samples, M_eff_ decreases substantially, from 10,400 to 7300 G for the sapphire/Py(40 nm) film and from 9600 to 7900 G for the Si/SiO_2_/Py(20 nm) film, whereas the TaN-encapsulated structure retains its value (10,300 G before and 10,700 G after magnetic annealing). A comparable reduction of the effective magnetization, occurring together with an increase in damping, has been reported for Py films that degraded during thermal annealing at 300 °C and above [23].

The linewidth also contains a frequency-independent contribution ΔH_0_ and a frequency-dependent contribution associated with the effective Gilbert damping α_eff_. For low-linewidth structures, the TaN/Py/TaN sample 2.4 exhibits α_eff_ = 0.0063 and ΔH_0_ = 4.2 Oe, compared with α_eff_ = 0.0073 and ΔH_0_ = 5.0 Oe for the uncapped 20 nm Py sample 2.1. After magnetic annealing, the TaN-encapsulated sample 3.2 retains a relatively low α_eff_ = 0.0055, although its ΔH_0_ increases to 6.7 Oe. These results indicate that the improved linewidth of the TaN-encapsulated structure cannot be attributed solely to differences in effective magnetization or effective Gilbert damping, suggesting that interface engineering also affects the extrinsic linewidth contribution.

The uncapped Py films were exposed to ambient air after deposition and during subsequent handling and measurements; therefore, the formation of a native oxide layer cannot be excluded. Although the magnetic annealing was carried out in a nitrogen atmosphere, the native oxide was already present before the thermal treatment. Such annealing can therefore promote its growth and reorganization and drive interdiffusion at the Py/oxide interface, rather than initiating oxidation.

Native oxidation of Py has been reported to produce a surface oxide consisting of NiO and Fe oxide [26]. A comparison of metallic and oxide-terminated interfaces on Py showed that a metallic capping layer yields purely Gilbert-like damping with negligible inhomogeneous broadening, whereas an oxidized surface introduces additional non-Gilbert contributions to the FMR linewidth [14]. In the present study, the TaN-encapsulated structure corresponds to the metallic-interface case, while the uncapped films develop a native oxide, which is consistent with their larger ΔH_0_ and pronounced post-annealing broadening. However, as no chemical analysis of the film surfaces was performed, its quantitative contribution to the measured linewidth cannot be disentangled from other effects such as surface roughness, interdiffusion, and interface-related magnetic inhomogeneity.

The surface topography of the samples was analyzed by AFM. The roughness data are shown in Figure 4d. The results demonstrate that the topography of the Py films depends not only on the deposition method and deposition temperature, but also on the film thickness and the presence of TaN buffer and capping layers.

The magnetron-sputtered films with thicknesses of 40 nm and 20 nm exhibited relatively low surface roughness, indicating that this deposition technique is capable of producing smooth, homogeneous thin films when appropriate process parameters are employed. By comparison, several samples prepared by electron-beam evaporation displayed higher roughness values. This behavior is likely related to differences in film growth dynamics, as well as variations in substrate preparation, deposition temperature, and the nature of the film–substrate interaction. Surface roughness has already been shown to affect linewidth broadening [13].

The AFM images in Figure 5 show one representative sample from each series and confirm that the TaN/Py/TaN structure has a smoother and more uniform topography than the uncapped 20 nm Py film, consistent with its lower S_a_ in Figure 4d and with the narrower FMR linewidth measured for this sample. Across the full sample set, however, the relation between roughness and linewidth is not one-to-one, indicating that interface quality rather than topography alone governs the extrinsic contribution [11,12,13,14].

Annealing in a static magnetic field was performed to investigate whether heat treatment could improve the magnetic resonance properties of the Py films. In principle, magnetic annealing may reduce internal stress, stabilize magnetic domains, and induce or strengthen uniaxial magnetic anisotropy [27]. However, the experimental results show that the effect of such annealing strongly depends on the presence of buffer and capping layers.

For the uncapped sapphire/Py (40 nm) sample, magnetic annealing led to a significant increase in the FMR linewidth. A similar deterioration was observed for the uncapped Si/SiO_2_/Py (20 nm) film. This broadening indicates that magnetic annealing did not improve the magnetic properties of these samples. Instead, it likely enhanced structural or magnetic inhomogeneity, possibly through changes in surface topography, stress redistribution, or interdiffusion at the film interfaces [12,13,14]. Thermal annealing of Py at temperatures up to 200 °C in ultrahigh vacuum has been reported to leave the Gilbert damping unchanged, with a drastic increase occurring only at 300 °C and above [23]. The broadening observed here for the uncapped films after magnetic annealing at 200 °C is therefore unlikely to originate from the thermal treatment alone, and points instead to degradation of the unprotected film surface.

In contrast, the TaN/Py/TaN sample retained a relatively narrow linewidth after magnetic annealing. This suggests that the TaN layers protect the Py film during heat treatment and help preserve its magnetic uniformity. The buffer and capping layers may reduce interdiffusion, stabilize the interfaces, and prevent significant roughness degradation. As a result, the annealed, TaN-encapsulated sample maintains favorable magnetic-resonance properties [8,14,17,18]. Since the samples were annealed under identical conditions, the contrast between the TaN-encapsulated and uncapped ones isolates the presence of the TaN layers as the decisive difference.

Thus, magnetic annealing alone is not sufficient to improve the dynamic magnetic properties of thin Py films. Its effect depends strongly on the initial film quality and interface stability. The best results are obtained when magnetic annealing is combined with appropriate interface engineering through buffer and capping layers.

The transmission spectra |S_21_| were simulated for selected non-annealed and annealed samples with the narrowest experimental linewidths, placed on a microstrip line on the substrate corresponding to each sample, for each deposition method and for the TaN-encapsulated structure. For every sample, the measured linewidth and the effective magnetization listed in Table 2 were used as input, so that the simulated spectra correspond to the experimentally determined magnetic parameters of the individual films.

The red curve in Figure 6a, corresponding to annealed sample 3.1, shows suppressed spin-wave features compared with the non-annealed sample 1.6 (black curve, prominent excitations). Both samples 1.6 and 3.1 are for the sapphire/Py(40 nm) structure. The increase in magnetic losses leads to stronger damping of the spin-wave modes and reduces their visibility in the transmission spectrum [8,9,28,29]. A similar tendency is observed for sample 3.3 (red curve in Figure 6b). After magnetic annealing, the spin-wave resonances become less pronounced compared with the non-annealed sample 2.1 (black curve).

In contrast, the spectra of samples 2.4 and 3.2 are similar (see Figure 6c). This indicates that the TaN-encapsulated Py film retains its spin-wave response after magnetic annealing. The weak influence of magnetic annealing on the simulated transmission spectrum agrees with the experimental FMR results, which show that the linewidth of the TaN-encapsulated structure remains relatively narrow after heat treatment.

Accordingly, films with lower magnetic losses (e.g., samples 1.6 and 2.1) show clearer resonant features in the calculated ∣S_21_∣ spectra, whereas films with broader resonance lines (e.g., samples 3.1 and 3.3) exhibit suppressed spin-wave signatures [10,28,29].

To demonstrate the possibility of spin-wave excitation in the studied Py films, the spatial distribution of the magnetic field component H_z_ was numerically calculated. The calculations were performed for sample 2.4 (denoted 3.2 after magnetic annealing), which had the narrowest linewidth in the experiment. The simulations were done in the xoy plane passing through the middle of the sample, in an external magnetic field of 280 Oe. The selected frequencies correspond to transmission minima in the calculated ∣S_21_∣ spectrum.

The calculated magnetic field component H_z_ spatial distributions are shown in Figure 7 for frequencies of 5.05, 5.48, 5.91, and 6.46 GHz. The results reveal standing spin-wave patterns across the film width. As the excitation frequency increases, the number of half-wavelengths fitting along the y-direction increases, from one at 5.05 GHz to seven at 6.46 GHz. This behavior is typical for quantized width modes in confined magnetic films [28,29,30]. Similar relationships between geometrical confinement, dynamic susceptibility, and the spatial profiles of spin-wave modes have been reported for elongated and stadium-shaped permalloy nanostructures [31,32].

Due to the symmetry of the microwave excitation with respect to the center of the film, only modes with an odd number of half-wavelengths across the width are efficiently excited [28,30]. Along the x-direction, the excitation is nearly in phase over the whole 50 µm length, so only the fundamental profile is excited there, and the mode index *n* therefore refers to the quantization across the 12 µm width. The distributions in Figure 7a–d correspond to *n* = 1, 3, 5, and 7 at 5.05, 5.48, 5.91, and 6.46 GHz, respectively—that is, only odd-index width modes appear, in agreement with the symmetry of the excitation. The simulated microstrip geometry therefore excites a discrete set of spin-wave modes in the Py film, and these numerical results support the conclusion that Py films with sufficiently low magnetic losses are suitable for spin-wave excitation in the gigahertz frequency range.

The experimental verification of these numerically predicted spin-wave excitation and propagation characteristics in the prepared Py films is planned for future work, enabling a direct assessment of the influence of deposition conditions and film quality on magnonic performance.

## 4. Conclusions

Thin permalloy (Py, Ni_80_Fe_20_) films with thicknesses of 20 and 40 nm were systematically investigated using different deposition conditions, interface structures, and thermal treatments. The results show that these technological parameters affect the FMR linewidth to different degrees. Several of them can produce a narrow linewidth, but only interface protection preserves it after thermal treatment.

Several process routes led to comparable minimum linewidths: approximately 23 Oe at 4 GHz for the best electron-beam-evaporated film on sapphire, approximately 22 Oe for the plasma-cleaned 40 nm sputtered film, and approximately 21 Oe for the 20 nm TaN/Py/TaN structure. These values lie within the measurement uncertainty of one another. Within the electron-beam series, substrate choice and surface preparation were decisive, whereas the deposition temperature and the magnetic field applied during growth had only a weak effect. Within the sputtered series, reducing the film thickness from 40 to 20 nm alone did not narrow the line.

The samples separate clearly only after post-deposition annealing in a static magnetic field (200 °C, 2 h). The uncapped Py films broaden substantially, indicating increased magnetic inhomogeneity, while the TaN/Py/TaN structure retains its narrow linewidth. TaN interface engineering therefore acts primarily by suppressing thermally activated extrinsic broadening and stabilizing the magnetic layer, rather than by lowering the as-deposited linewidth.

Numerical simulations of microstrip-driven spin-wave excitation illustrate that the measured FMR linewidth strongly influences the visibility and amplitude of spin-wave resonances. Films with reduced linewidth support well-defined standing spin-wave modes, whereas broader linewidths strongly suppress resonant features in the microwave transmission spectra.

Overall, these results show that low magnetic losses in Py films can be reached through several combinations of deposition route, substrate preparation and film thickness, whereas retaining them through subsequent thermal processing requires interface protection. The demonstrated optimization route offers a practical approach for preparing Py structures suitable for magnonic and spintronic devices operating in the gigahertz frequency range.

## Figures and Tables

**Figure 1:**
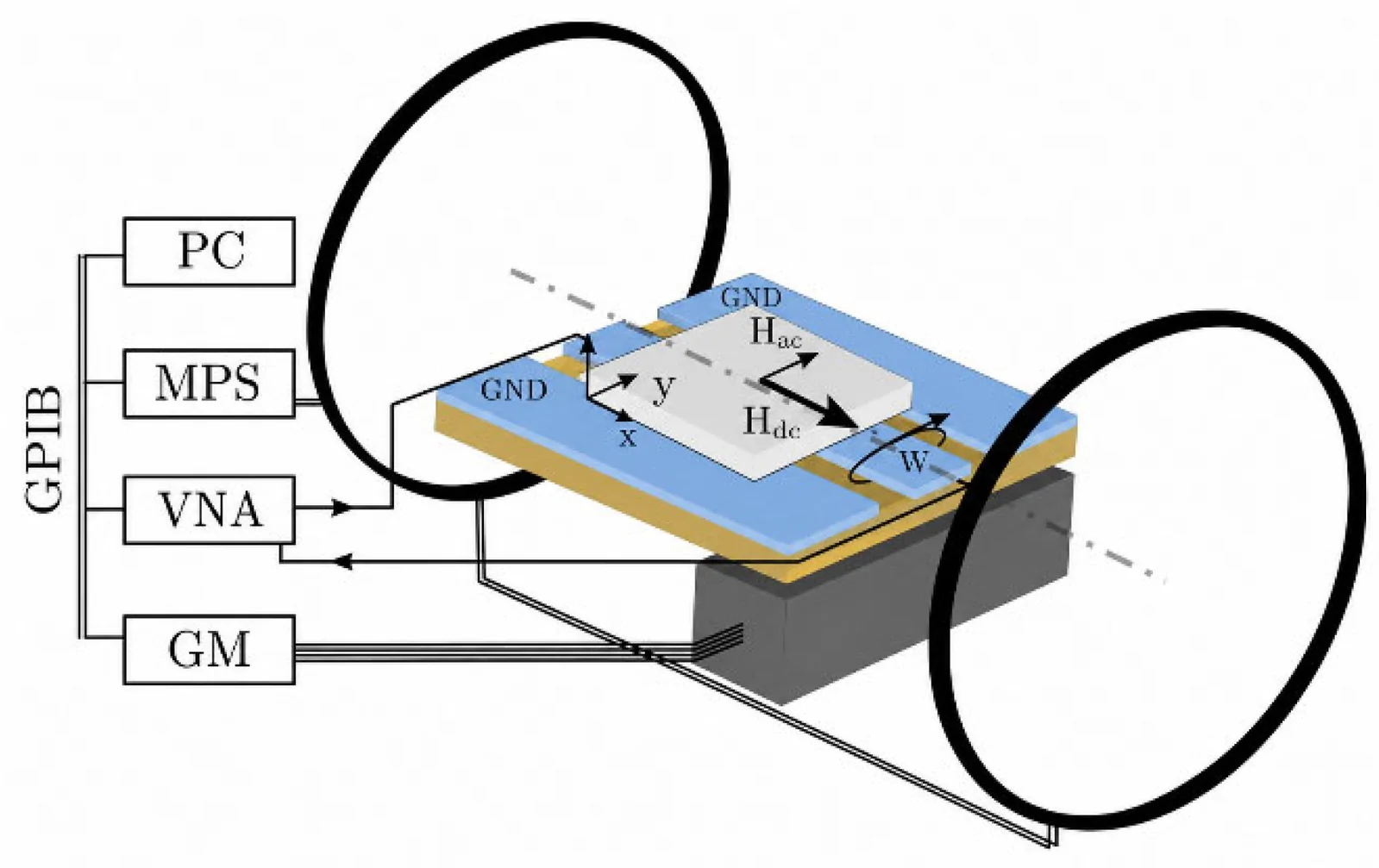
Scheme of the experimental setup used for broadband VNA-FMR measurements.

**Figure 2:**
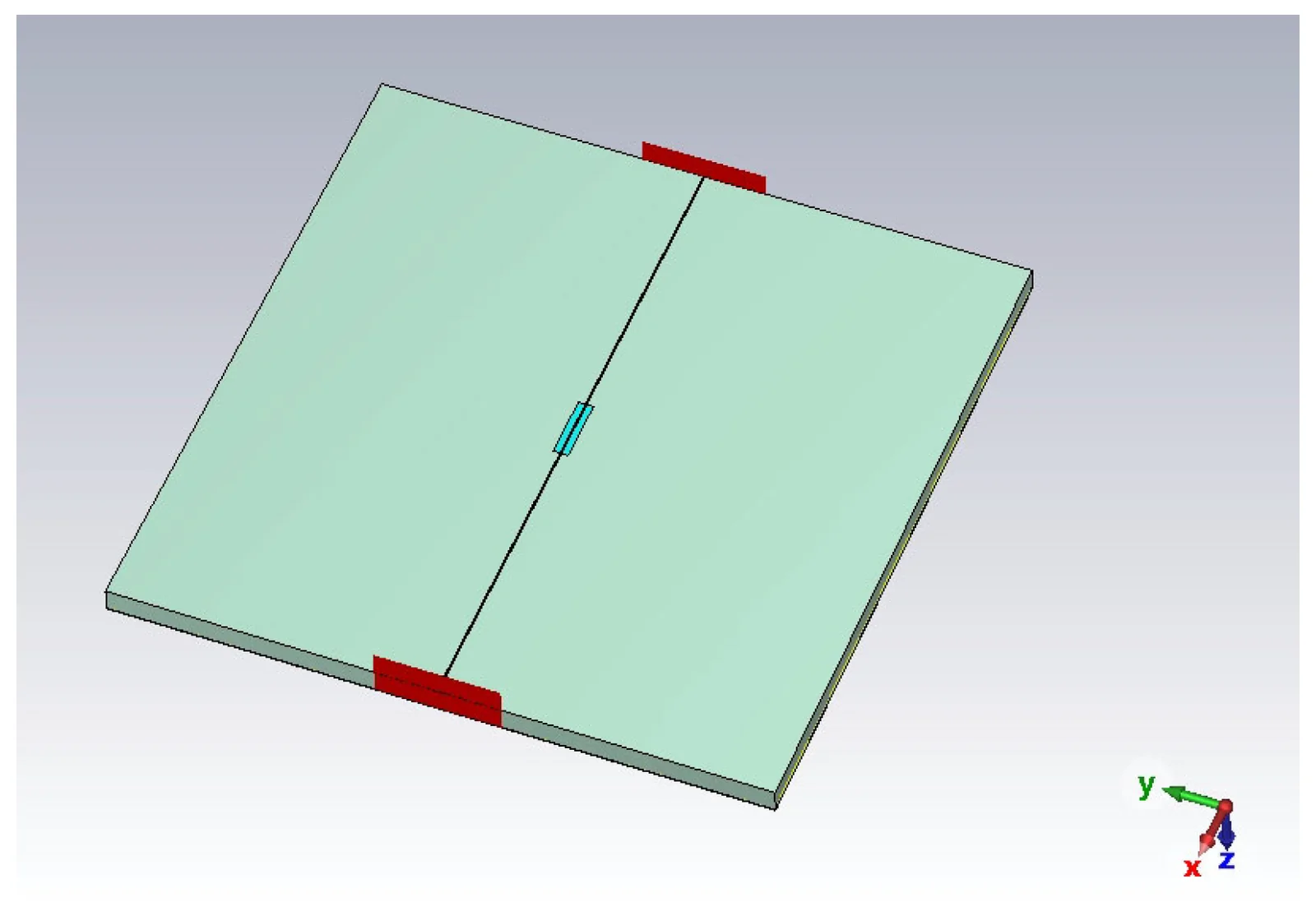
Permalloy film on a silicon or sapphire substrate placed on a microstrip line.

**Figure 3:**
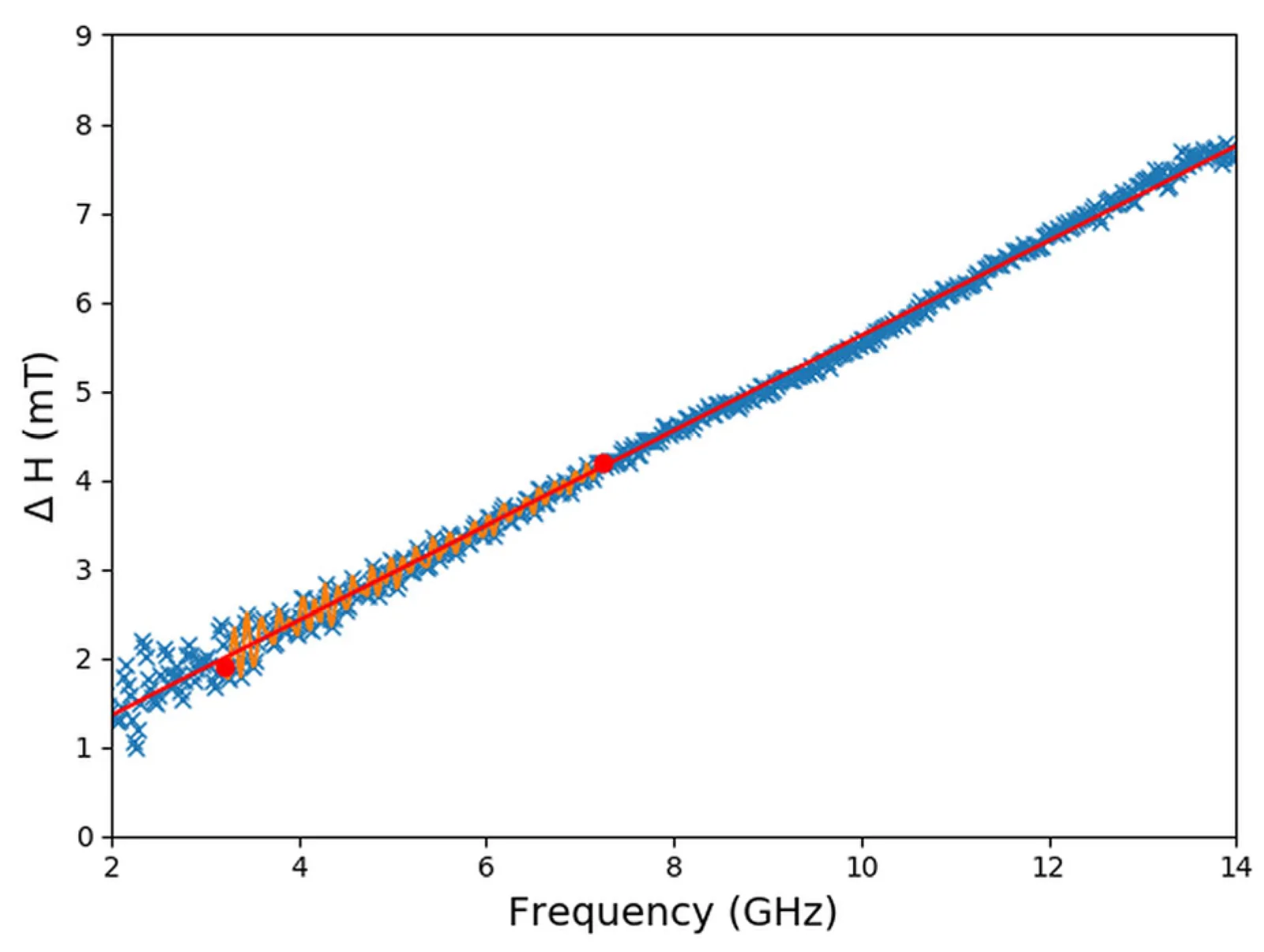
Frequency dependence of the linewidth ΔH(f) for sample 1.6 over the range 2–14 GHz, together with the linear fit. Blue crosses: experimental data measured for the sample with both positive and negative magnetic-field sweeps. Red line: linear fit to the data over the whole range.

**Figure 4:**
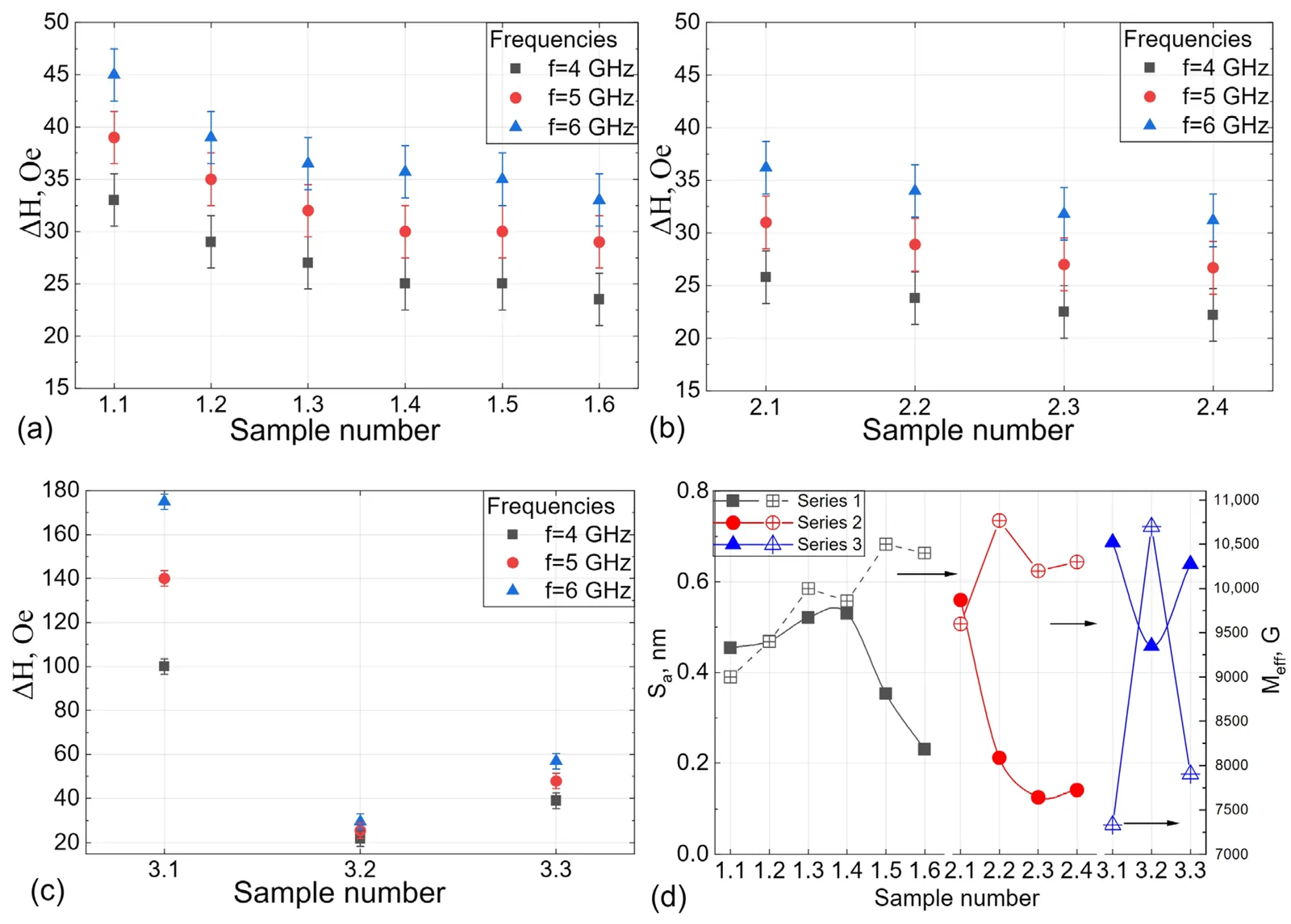
Ferromagnetic resonance linewidth as full width at half maximum for samples of series 1 (**a**), series 2 (**b**) and series 3 (**c**), with the corresponding uncertainties. In panel (**d**), filled symbols and the left axis show the average surface roughness S_a_ of the Py samples from all series, while open symbols and the right axis show the effective magnetization M_eff_; the arrows point towards the corresponding axis.

**Figure 5:**
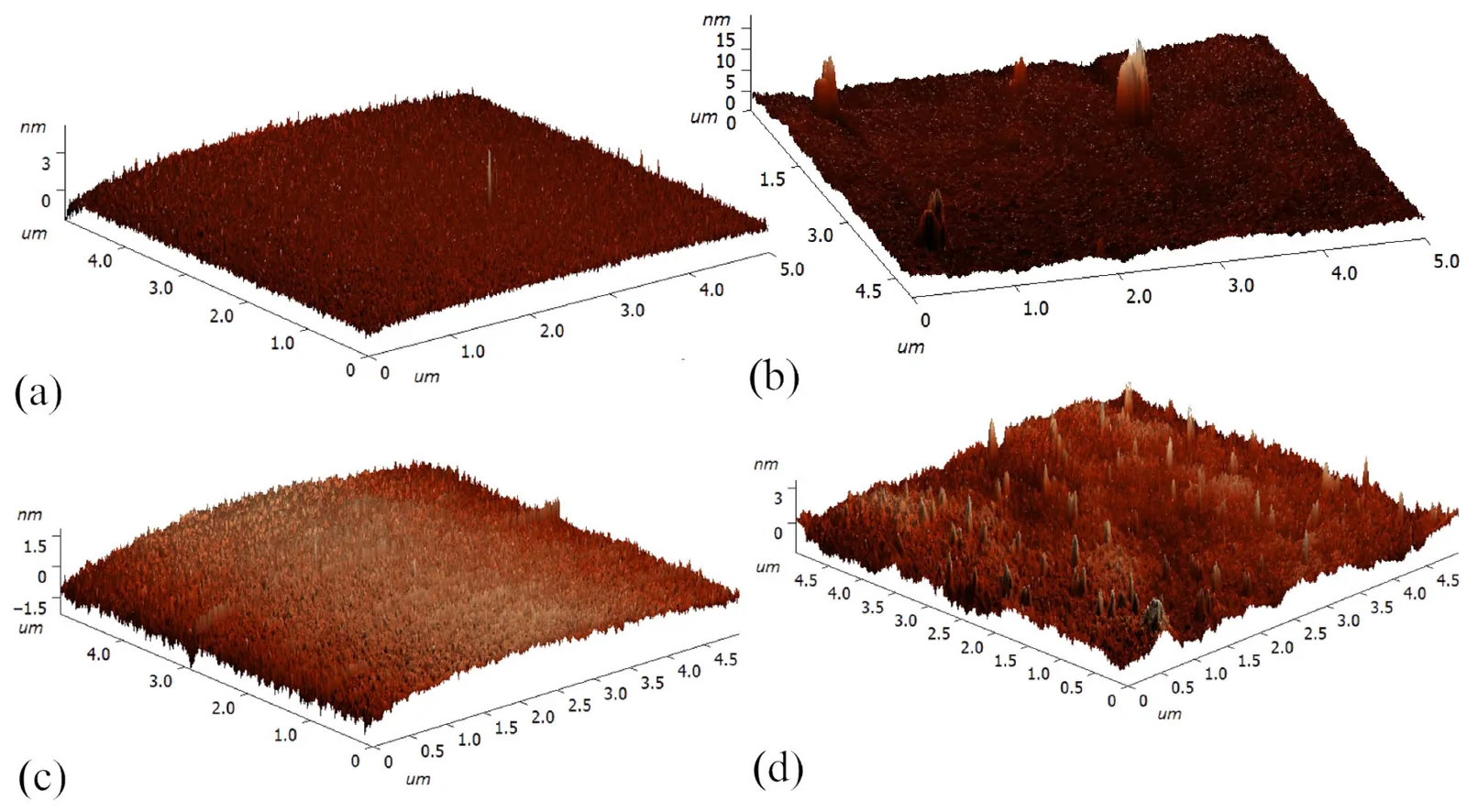
AFM images of the surface topography of sample 1.6 (**a**), sample 2.1 (**b**), sample 2.4 (**c**), and sample 3.2 (**d**).

**Figure 6:**
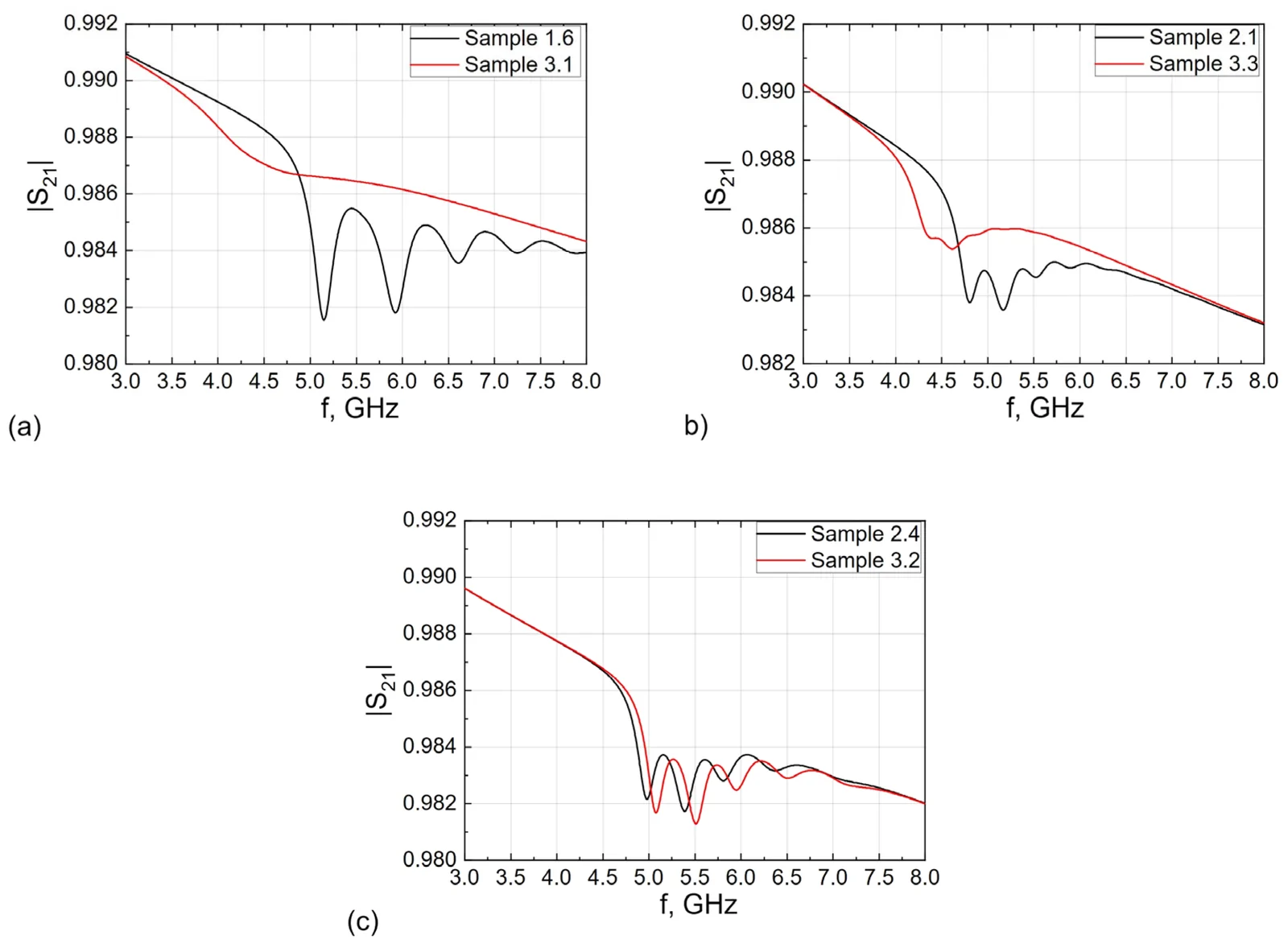
Simulated transmission coefficient spectra ∣S_21_∣ for sample 1.6 and annealed sample 3.1 (**a**), sample 2.1 and annealed sample 3.3 (**b**), and sample 2.4 and annealed sample 3.2 (**c**).

**Figure 7:**
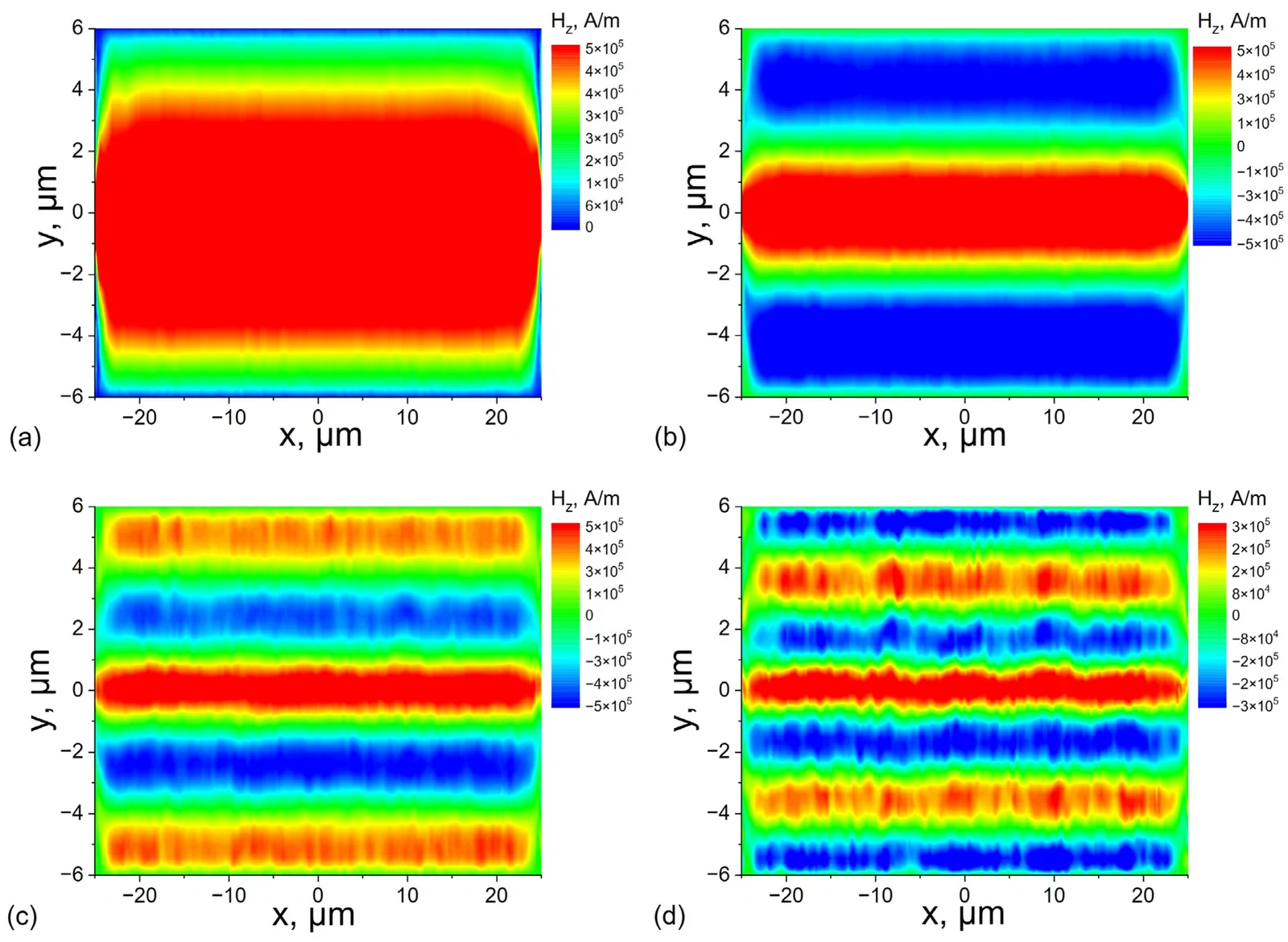
Numerically calculated spatial distribution of the magnetic field component H_z_ in the xoy plane passing through the middle of sample 3.2 in an external magnetic field of 280 Oe at 5.05 GHz (**a**), 5.48 GHz (**b**), 5.91 GHz (**c**), and 6.46 GHz (**d**).

**Table 1:** Studied Py thin-film samples.

Series/Sample	Structure	Deposition Method/Treatment	Deposition or Annealing Conditions	Applied Field
1.1	Si/SiO_2_/Py(40 nm)	Electron-beam evaporation	200 °C; manual cut	-
1.2	Si/SiO_2_/Py(40 nm)		Room temperature; laser cut	magnetic shielding during deposition
1.3	Si/SiO_2_/Py(40 nm)		Room temperature; laser cut	400 Oe
1.4	Si/SiO_2_/Py(40 nm)		Room temperature; manual cut	-
1.5	Si/SiO_2_/Py(40 nm)		Plasma cleaning; 200 °C; manual cut	-
1.6	Sapphire/Py(40 nm)		Plasma cleaning; room-temperature; laser cut	-
1.7	Si/SiO_2_/Py(40 nm)		Plasma cleaning; 400 °C; laser cut	-
2.1	Si/SiO_2_/Py(20 nm)	Magnetron sputtering	Room temperature; manual cut	-
2.2	Si/SiO_2_/Py(40 nm)			-
2.3	Si/SiO_2_/Py(40 nm)		Plasma cleaning; room temperature; manual cut	-
2.4	Si/SiO_2_/TaN(2–3 nm)/ Py(20 nm)/TaN(2–3 nm)		Room temperature; manual cut	-
3.1	Sapphire/Py(40 nm)	Electron-beam evaporation, then magnetic annealing	200 °C	400 Oe (2 h)
3.2	Si/SiO_2_/TaN(2–3 nm)/ Py(20 nm)/TaN(2–3 nm)	Magnetron sputtering, then magnetic annealing		
3.3	Si/SiO_2_/Py(20 nm)			

**Table 2:** Lateral correlation lengths (L_x_ and L_y_), surface roughness (S_q_, RMS), effective magnetization (M_eff_), effective Gilbert damping (α_eff_), and inhomogeneous linewidth (ΔH_0_) of the investigated samples.

Series/Sample	S_q_ (nm)	L_x_ (nm)	L_y_ (nm)	M_eff_ (G)	ΔH_0_, Oe	Gilbert Damping Parameter α_eff_
1.1	0.579	107	7.88	9000	9.0	0.0084
1.2	0.865	79.4	53.1	9400	9.33	0.0070
1.3	0.738	362	282	10,000	8.08	0.0067
1.4	0.697	283	7.91	9860	3.48	0.0081
1.5	0.419	479	365	10,500	5.0	0.0070
1.6	0.282	22.4	7.18	10,400	4.75	0.0067
1.7	—	—	—	—	—	—
2.1	0.772	342	280	9600	5.0	0.0073
2.2	0.260	18.8	6.93	10,770	3.4	0.0072
2.3	0.152	34.3	19.8	10,200	3.85	0.0065
2.4	0.183	29.6	19.8	10,300	4.2	0.0063
3.1	0.940	29.5	31.1	7300	0 ^a^	0.0391
3.2	0.587	284	295	10,700	6.7	0.0055
3.3	1.159	166	146	7900	3.15	0.0126

^a^ For sample 3.1, an unconstrained linear fit of ΔH(f) yields a negative intercept, which is unphysical. Therefore, ΔH_0_ was set to zero, and α_eff_ was obtained from a fit through the origin. The very large broadening of this sample indicates that its linewidth is not described by a simple Gilbert-type linear dependence, as expected when two-magnon scattering and local inhomogeneity dominate [5,11,23]. The tabulated α_eff_ should therefore be read as an upper-bound effective value rather than a Gilbert damping parameter. “—” indicates that the corresponding parameter was not determined.

## Data Availability

Data are contained within the article or Supplementary Materials.

## Supplementary Materials

The following supporting information can be downloaded at https://www.mdpi.com/article/10.3390/nano16191209/s1, Figure S1: Frequency dependence of the linewidth ΔH(f) for all samples over the range 3–9 GHz, with sample number indicated in each panel.

## Abbreviations

The following abbreviations are used in this manuscript:

AFM	Atomic force microscopy
DPPH	2,2-diphenyl-1-picrylhydrazyl
FMR	Ferromagnetic resonance
Py	Permalloy
VNA	Vector network analyzer
