# Supplementary material for: Magnetic Resonance Properties of Thin Py Films Suitable for Excitation of Spin Waves
Source: Nanomaterials (Basel). 2026 Sep 24;16(19):1209. doi: 10.3390/nano16191209 (PMC13666942; doi:10.3390/nano16191209)
Supplement: Supplementary file 1 [file nanomaterials-16-01209-s001.zip › nanomaterials-4556857-supplementary.pdf]

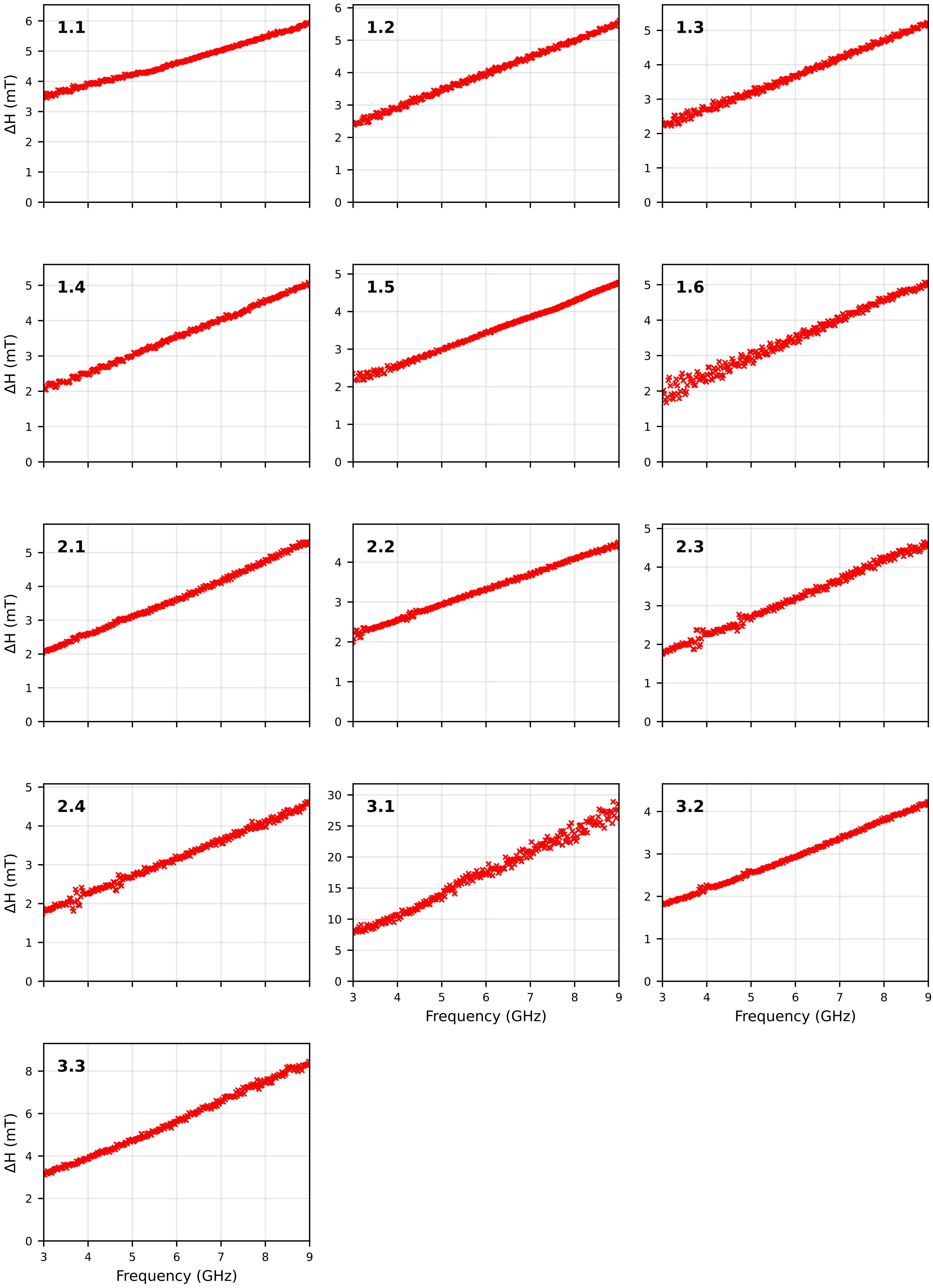

Figure S1. Frequency dependence of the linewidth ΔH(f) for all samples over the range 3–9 GHz, with sample number indicated in each panel.
